# Celebrating a Century of Research in Behavioral Genetics

**DOI:** 10.1007/s10519-023-10132-3

**Published:** 2023-01-20

**Authors:** Robert Plomin

**Affiliations:** Institute of Psychiatry, Psychology and Neuroscience, King’s College London, London, UK

**Keywords:** Quantitative genetics, Molecular genetics, Genome-wide association, Polygenic scores, Intelligence

## Abstract

A century after the first twin and adoption studies of behavior in the 1920s, this review looks back on the journey and celebrates milestones in behavioral genetic research. After a whistle-stop tour of early quantitative genetic research and the parallel journey of molecular genetics, the travelogue focuses on the last fifty years. Just as quantitative genetic discoveries were beginning to slow down in the 1990s, molecular genetics made it possible to assess DNA variation directly. From a rocky start with candidate gene association research, by 2005 the technological advance of DNA microarrays enabled genome-wide association studies, which have successfully identified some of the DNA variants that contribute to the ubiquitous heritability of behavioral traits. The ability to aggregate the effects of thousands of DNA variants in polygenic scores has created a DNA revolution in the behavioral sciences by making it possible to use DNA to predict individual differences in behavior from early in life.

## Introduction

Although the history of heredity and behavior can be traced back to ancient times (Loehlin 2009), the first human behavioral genetic research was reported in the 1920s, which applied quantitative genetic twin and adoption designs to assess genetic influence on newly developed measures of intelligence. The 1920s also marked the beginning of single-gene research that led to molecular genetics. The goal of this review is to outline 100 years of progress in quantitative genetic and molecular genetic research on behavior, a whistle-stop tour of a few of the major milestones in the journey. The review focuses on human research even though non-human animal research played a major role in the first 50 years (Maxson 2007). It uses intelligence as a focal example because intelligence was the target of much human research, even though a similar story could be told for other areas of behavioral genetics such as psychopathology.

### The Two Worlds of Genetics

The most important development during this century of behavioral genetic research has been the synthesis of the two worlds of genetics, quantitative genetics and molecular genetics. Quantitative genetics and molecular genetics both have their origins in the 1860s with Francis Galton (Galton 1865, 1869) and Gregor Mendel (Mendel 1866), respectively. Not much happened until the 1900s when Galton’s insights led to methods to study genetic influence on complex traits and when Mendel’s work was re-discovered. The two worlds clashed as Mendelians looked for 3:1 segregation ratios indicative of single-gene traits, whereas Galtonians assumed that Mendel’s laws of heredity were specific to pea plants because they knew that complex traits are distributed continuously.

Antipathy between the two worlds of genetics followed because of the different goals of Mendelians and Galtonians. Mendelians, the predecessors of molecular geneticists, wanted to understand how genes work, which led to the use of induced mutations and a focus on dichotomous traits that were easily assessed such as physical characteristics rather than behavioral traits. In contrast, Galtonians, whose descendants are quantitative geneticists, used genetics as a tool to understand the etiology of naturally occurring variation in complex traits selected for their intrinsic interest and importance, with behavioral traits, especially intelligence, high on the list. The resolution to the conflict could be seen in Ronald Fisher’s 1918 paper, which showed that Mendelian inheritance is compatible with quantitative traits if the assumption is made that several genes affect a trait (Fisher 1918). Nonetheless, the two worlds of genetics went their own way for most of the century.

**Fig. 1:**
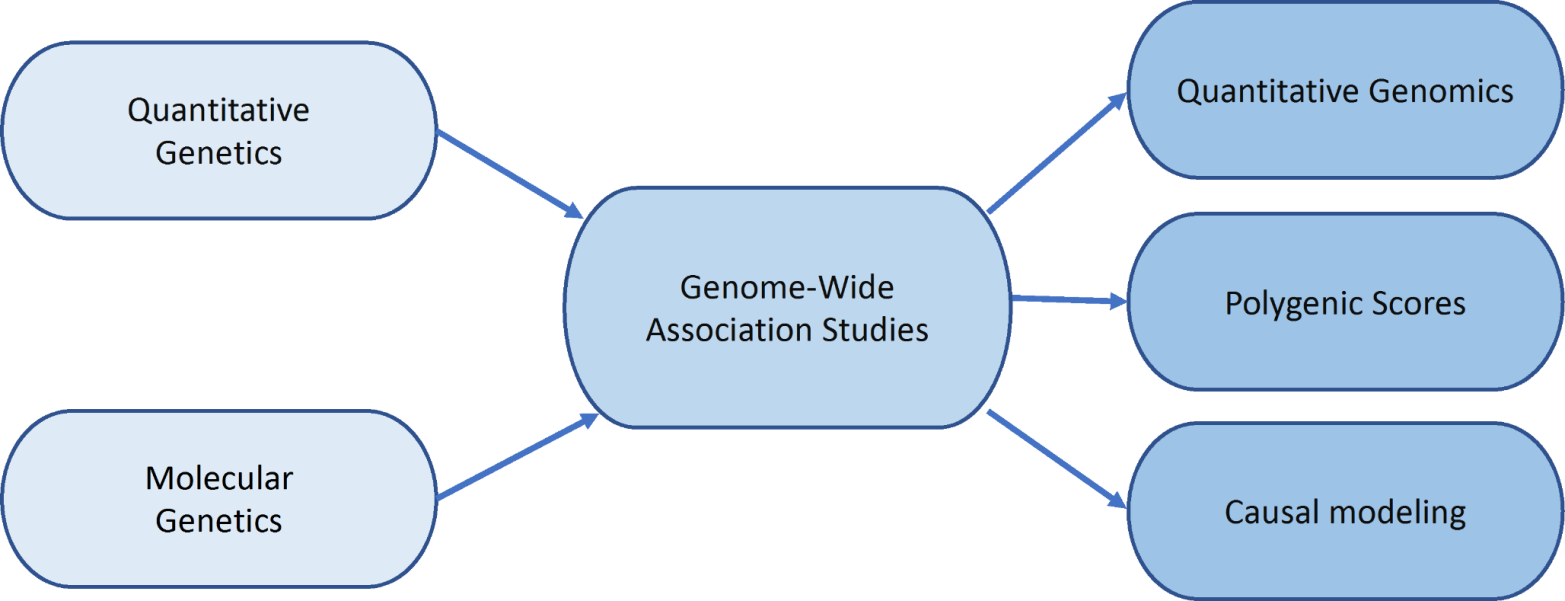
Synthesis of the two worlds of genetics: from behavioral genetics to behavioral genomics.

The synthesis of the two worlds of genetics began in the 1980s with the technological advances of DNA sequencing, polymerase chain reaction, and DNA microarrays that enabled genome-wide association (GWA) studies of complex traits. In addition to finding DNA variants associated with complex traits, GWA genotypes led to three far-reaching advances in genetic research. First, GWA genotypes were used to estimate directly the classical quantitative genetic parameters of heritability and genetic correlation, which could be called *quantitative genomics*. Second, the results of GWA studies were used to create polygenic scores that predict individual differences for complex traits. Third, GWA genotypes facilitated new approaches to causal modeling of the interplay between genes and environment. Together, when applied to behavioral traits, these advances could be called *behavioral genomics*. This synthesis of the two worlds of genetics, the journey from behavioral genetics to behavioral genomics, is the overarching theme of this whistle-stop tour celebrating a century of research in behavioral genetics. (See Fig. 1.) The itinerary begins with milestones in quantitative genetics and then molecular genetics, concluding with behavioral genomics.

### Quantitative Genetics

The first 50 years of quantitative genetic research, from 1920 to 1970, started off well with family studies (Jones 1928; Thorndike 1928), twin studies (Holzinger 1929; Lauterbach 1925; Merriman 1924; Tallman 1928) and adoption studies (Burks 1928; Freeman et al. 1928) using the recently devised IQ test. However, this nascent research was squelched with the emergence of Nazi eugenic policies (McGue 2008). The void was filled with behaviorism (Watson 1930), which led to environmentalism, the ‘blank slate’ view that we are what we learn (Pinker 2003).

Nonetheless, a few studies of IQ appeared in the 1930 and 1940 s, such as the first study of identical twins reared apart (Newman et al. 1937) and the first adoption study that assessed birth parents (Skodak and Skeels 1949). Both indicated substantial genetic influence on IQ, as did a review of all available IQ data (Woodworth 1941).

In 1960, the field-defining book, *Behavior Genetics* (Fuller and Thompson 1960), was published. It mostly reviewed research on nonhuman animals. In their preface, the authors noted that “we considered omitting human studies completely” (p. vi); even their chapter on cognitive abilities primarily reviewed nonhuman research. An earlier influential review began by saying, “In the writer’s opinion, the genetics of behavior must be worked out on species that can be subjected to controlled breeding. At the present time this precludes human subjects” (Hall 1951).

In 1963, a milestone review was published in *Science* of 52 family, twin and adoption studies of IQ (Erlenmeyer-Kimling and Jarvik 1963). Although the studies were very small by modern standards and heritability was not calculated, the average results from the different designs suggested substantial heritability. For example, the average MZ and DZ twin correlations were 0.87 and 0.53, respectively, suggesting a heritability of 68%. However, despite being published in *Science*, the paper was largely ignored; it was cited only 22 times in five years.

The pace of behavioral genetic research picked up in the 1960s, once again primarily research on non-human animals (Lindzey et al. 1971; McClearn 1971), although some twin studies on cognitive abilities were also published (Nichols 1965; Schoenfeldt 1968). However, the first 50 years of quantitative genetic research ended badly with the publication in 1969 of Arthur Jensen’s paper, *How Much Can We Boost IQ and Scholastic Achievement?* (Jensen 1969). The paper touched on ethnic differences, which made it one of the most controversial papers in the behavioral sciences, with 900 citations in the first five years and more than 6200 citations in total.

1970 was a watershed year marking the second 50 years of behavioral genetic research. It was the year that the Behavior Genetics Association was launched and the first issue of its journal, *Behavior Genetics*, was published. Another 1970 milestone was the publication of the foundational paper for model-fitting analysis of quantitative genetic designs (Jinks and Fulker 1970).

The 1970s and 1980s yielded most of the major discoveries for quantitative genetics as applied to behavioral traits, discoveries that are listed as landmarks in the following paragraphs. Nonetheless, in the aftermath of Jensen’s 1969 paper, behavioral genetic research, especially on intelligence, was highly controversial (Scarr and Carter-Saltzman 1982). Most notably, Leon Kamin severely criticized the politics as well as science of behavioral genetic research on intelligence in his book, *The Science and Politics of I.Q.* (Kamin 1974). He concluded that “There exist no data which should lead a prudent man to accept the hypothesis that I.Q. test scores are in any degree heritable” (p. 1). The book was cited more than 2000 times and stoked antipathy towards genetic research. It also impugned the motivation of genetic researchers, saying that they are ‘committed to the view that those on the bottom are genetically inferior victims of their own immutable defects’ (p. 2).

#### All Traits are Heritable

Despite this hostility, genetic research grew exponentially in the 1970s and created a seismic shift from the prevailing view that behavioral traits like intelligence are not “in any degree heritable”. In 1978, a review of 30 twin studies of intelligence yielded an average heritability estimate of 46% (Nichols 1978). Moreover, the conclusion began to emerge that all traits show substantial heritability. This conclusion, which has been called the first law of behavioral genetics (Turkheimer 2000), was first observed in 1976 in a twin study of cognitive data for 3000 twin pairs, which also included extensive data on personality and interests for 850 twin pairs (Loehlin and Nichols 1976). The authors noted “the curious uniformity of identical-fraternal differences both within and across trait domains” (p. 89). A 2015 meta-analysis of all published twin studies showed that behavioral traits are about 50% heritable on average (Polderman et al. 2015). Demonstrating the ubiquitous importance of genetics was the fundamental accomplishment of behavioral genetics.

#### No Traits are 100% Heritable

The flip side of the finding of 50% heritability was just as important: no traits are 100% heritable. It is ironic that, after a century of environmentalism, genetic research provided the strongest evidence for the importance of the environment; previous environmental research was confounded because it ignored genetics. Moreover, investigating environmental influences in genetically sensitive designs led to two of the most important discoveries about the environment: nonshared environment and the nature of nurture.

#### Nonshared Environment

Quantitative genetic research showed that environmental influences work very differently from the way they were assumed to work. A second discovery by Loehlin and Nichols (1976) was that salient environmental influences are not shared by twins growing up in the same family: “Environment carries substantial weight in determining personality – it appears to account for at least half the variance – but that environment is one for which twin pairs are correlated close to zero” (p. 92). This phenomenon has come to be known as nonshared environment (Plomin and Daniels 1987).

Loehlin and Nichols suggested that cognitive abilities are an exception to the rule that environmental influences make children in a family different from, not similar to, one another. Their twin study suggested that about 25% of the variance of cognitive abilities could be attributed to shared environment. A direct test of shared environmental influence is the correlation between adoptive siblings, genetically unrelated children adopted into the same family. Seven small studies of adoptive siblings yielded an average IQ correlation of 0.25, which seemed to precisely confirm the twin estimate (McGue et al. 1993).

However, in 1978, a study of 100 pairs of adoptive siblings reported an IQ correlation of -0.03 (Scarr and Weinberg 1978). This is a good example of the progressive nature of behavioral genetic research (Urbach 1974). Scarr and Weinberg noted that previous studies involved children, whereas theirs was the first study of post-adolescent adoptive siblings aged 16 to 22, and they hypothesized that the effect of shared environmental influence on cognitive development diminishes after adolescence as young adults make their own way in the world. Their hypothesis was confirmed in two additional studies of post-adolescent adoptive siblings that yielded an average IQ correlation of -0.01 (McGue et al. 1993). Evidence that shared environmental influence declines after adolescence to negligible levels for cognitive abilities has also emerged from twin studies (Briley and Tucker-Drob 2013; Haworth et al. 2010). However, one of the biggest mysteries about nonshared environment remains: what are these environmental influences that make children growing up in the same family so different (Plomin 2011)?

#### The Nature of Nurture

Another milestone was the revelation that environmental measures widely used in the behavioral sciences, such as parenting, social support, and life events, show genetic influence (Plomin and Bergeman 1991), with heritabilities of about 25% on average (Kendler and Baker 2007). This finding emerged in the 1980s as measures of the environment were included in quantitative genetic designs, which also led to the discovery that associations between environmental measures and psychological traits are significantly mediated genetically (Plomin et al. 1985). The nature of nurture is one of the major directions for research in behavioral genomics, as discussed later.

#### Heritability Increases During Development

Another milestone in the 1970s was the Louisville Twin Study in which mental development of 500 pairs of twins was assessed longitudinally and showed that the heritability of intelligence increases from infancy to adolescence (Wilson 1983). In light of the replication crisis in science (Ritchie 2021), a cause for celebration is that this counterintuitive finding of increasing heritability of intelligence – from about 40% in childhood to more than 60% in adulthood -- has consistently replicated, as seen in cross-sectional (Haworth et al. 2010) and longitudinal (Briley and Tucker-Drob 2013) mega-analyses.

#### Pleiotropy

In 1977, a landmark paper showed how univariate analysis of variance can be extended to multivariate analysis of covariance in a model-fitting framework (Martin and Eaves 1977). They applied their approach to cognitive abilities and found an average genetic correlation of 0.52, indicating that many genes affect diverse traits, called *pleiotropy*. Subsequent studies also yielded genetic correlations greater than 0.50 between diverse cognitive abilities (Plomin and Kovas 2005).

#### Summary

In the 1970s and 1980s, bigger and better studies made most of the major quantitative genetic discoveries, going far beyond merely estimating heritability. But it was not all smooth sailing. Most notably, *The Bell Curve* resurrected many of the issues that followed Jensen’s 1969 paper (Herrnstein and Murray 1996). Nonetheless, by the 1990s, quantitative genetic research had convinced most scientists of the importance of genetics for behavioral traits, including intelligence (Snyderman and Rothman 1990). One symbol of this change was that the 1992 Centennial Conference of the American Psychological Association chose behavioral genetics as one of two themes that best represented the past, present, and future of psychology (Plomin and McClearn 1993). Then, just as quantitative genetic discoveries began to slow, the synthesis with molecular genetics began, which led to the DNA revolution and behavioral genomics.

### Molecular Genetics

During its first 50 years, molecular genetics focused on single-gene disorders. In 1933, a Nobel prize was awarded to Thomas Hunt Morgan for mapping genes responsible for single-gene mutations in fruit flies (Morgan et al. 1923), but human mapping was stymied because only a few single-gene markers such as blood types were available – variants in DNA itself were not available for another fifty years. Research on single-gene effects discovered in pedigree studies only incidentally involved behavioral traits. For example, phenylketonuria, the most common single-gene metabolic disorder, was discovered in 1934 (Folling 1934) and shown to be responsible for 1% of the population institutionalized for severe intellectual disability.

In the 1940s, it became clear that DNA is the mechanism of heredity, culminating in the most famous paper in biology which proposed the double-helix structure of DNA (Watson and Crick 1953). An important milestone for human behavioral genetics was the discovery in 1959 that the most common form of intellectual disability, Down syndrome, was due to a trisomy of chromosome 21 (Lejeune et al. 1959).

In 1961, the genetic code was cracked showing that three-letter sequences of the four-letter alphabet of DNA coded for the 20 amino acids (Crick et al. 1961). Just as with quantitative genetics, the 1970s was a watershed decade that ushered in the second 50 years, the genomics era.

#### The Genomics Era

The era of genomics began in the 1970s when methods were developed to sequence DNA’s nucleotide bases (Sanger et al. 1977). In 2003, fifty years after the discovery of the double helix structure of DNA, the Human Genome Project identified the sequence of 92% of the three billion nucleotide bases in the human genome (Collins et al. 2003).

In the 1980s, the first common variants in DNA itself were discovered, restriction fragment length polymorphisms (RFLPs) (Botstein et al. 1980). RFLPs enabled linkage mapping for single-gene disorders and were the basis for DNA fingerprinting, which revolutionized forensics (Jeffreys 1987). Polymerase chain reaction (PCR) was also developed which facilitated genotyping by rapidly amplifying DNA fragments (Mullis et al. 1986). In the 1980s, these developments increased the pace of linkage mapping of single-gene disorders, many of which had cognitive consequences, such as phenylketonuria (Woo et al. 1983) and Huntington disease (Gusella et al. 1983). In the 1990s, DNA sequencing revealed thousands of single-nucleotide polymorphisms (SNPs), the most common DNA variant (Collins et al. 1997).

In the 1990s, linkage was also attempted for complex traits that did not show single-gene patterns of transmission, such as reading disability (Cardon et al. 1994), but these were unsuccessful because linkage, which traces chromosomal recombination between disease genes and DNA variants within families, is unable to detect small effect sizes (Plomin et al. 1994). Researchers then pivoted towards allelic association in unrelated individuals, which is much more powerful in detecting DNA variants of small effect size. An early example of association was an allele of the *apolipoprotein E* gene on chromosome 19 that was found in 40% of individuals with late-onset Alzheimer disease as compared to 15% in controls (Corder et al. 1993).

The downside of allelic association is that an association can only be detected if a DNA variant is itself the functional gene or very close to it. For this reason, and because genotyping each DNA variant was slow and expensive, the 1990s became the decade of candidate gene studies in which thousands of studies reported associations between complex behavioral traits and a few ‘candidate’ genes, typically neurotransmitter genes thought to be involved in behavioral pathways. However, these candidate-gene associations failed to replicate because these studies committed most of the sins responsible for the replication crisis (Ioannidis 2005). For example, when 12 candidate genes reported to be associated with intelligence were tested in three large samples, none replicated (Chabris et al. 2012).

#### Genome-wide Association

In 1996, an idea emerged that was the opposite of the candidate-gene approach: using thousands of DNA variants to systematically assess associations across the genome in large samples of unrelated individuals (Risch and Merikangas 1996). However, genome-wide association (GWA) seemed a dream because genotyping was slow and expensive.

The problem of genotyping each DNA variant in large samples was solved in the 2000s by the commercial availability of DNA microarrays, called *SNP chips*, which genotype hundreds of thousands of SNPs for an individual quickly, accurately, and inexpensively. SNP chips paved the way for GWA analyses. In 2007, the first major GWA analysis included 2000 cases for each of seven major disorders and compared SNP allele frequencies for these cases with controls (The Wellcome Trust Case Control Consortium 2007). Replicable associations were found but they were few in number and extremely small in effect size. Hundreds of GWA reports appeared in the next decade with similarly small effect sizes across the behavioral and biological sciences (Visscher et al. 2017), including cognitive traits such as educational attainment (Rietveld et al. 2013) and intelligence in childhood (Benyamin et al. 2014) and adulthood (Davies et al. 2011).

These GWA studies led to the realization that the biggest effect sizes were much smaller than anyone anticipated. For case-control studies, risk ratios were less than 1.1, and for dimensional traits, variance explained was less than 0.001. This meant that huge sample sizes would be needed to detect these miniscule effects, and thousands of these associations would be needed to account for heritability, which is usually greater than 50% for cognitive traits. Ever larger GWA samples scooped up more of these tiny effects. Most recently, a GWA meta-analysis with a sample size of 3 million netted nearly four thousand independent significant associations after correction for multiple testing, but the median effect size of these SNPs accounted for less than 0.0001 of the variance (Okbay et al. 2022).

A century after Fisher’s 1918 paper, the discovery of such extreme polygenicity (Boyle et al. 2017; Visscher et al. 2021) was a turning point in the voyage from behavioral genetics to behavioral genomics. GWA genotypes brought the two worlds of genetics together by making it possible to use GWA genotypes to create three sets of tools to investigate highly polygenic traits: quantitative genomics, polygenic scores, and causal modeling (see Fig. 1). When applied to behavioral traits, these tools constitute the new field of behavioral genomics.

### Quantitative Genomics

What good are SNP associations that account for such tiny effects? The molecular genetic goal of tracking effects from genes to brain to behavior is daunting when the effects are so small. However, in contrast to this bottom-up approach from genes to behavior, the top-down perspective of behavioral genetics answered this question by using GWA genotypes to estimate quantitative genetic parameters of heritability and genetic correlations, which could be called *quantitative genomics*. The journey picked up speed as quantitative genomics led to three new milestones.

*Genome-wide Complex Trait Analysis (GCTA).* In 2011, the first new method was devised to estimate heritability and genetic correlations since twin and adoption designs in the early 1900s. GCTA (originally called GREML) uses GWA genotypes for large samples of unrelated individuals to compare overall SNP similarity to phenotypic similarity pair by pair for all pairs of individuals (Yang et al. 2011). The extent to which SNP similarity explains trait similarity is called SNP heritability because it is limited to heritability estimated by the SNPs on the SNP chip. Genetic correlations are estimated by comparing each pair’s SNP similarity to their cross-trait phenotypic similarity.

SNP heritability estimates are about half the heritability estimated by twin studies (Plomin and von Stumm 2018). This ‘missing heritability’ occurs because SNP heritability is limited to the common SNPs genotyped on current SNP chips, which also creates a ceiling for discovery in GWA research. Most SNPs are not common, and rare SNPs appear to be responsible for much of the missing heritability, at least for height (Wainschtein et al. 2022). Importantly, quantitative genomic estimates of genetic correlations are not limited in this way and thus provide estimates of genetic correlations similar to those from twin studies (Trzaskowski et al. 2013).

*Linkage Disequilibrium Score (LDSC) Regression.* In 2015, a second quantitative genomic method, LDSC, was published which estimates heritability and genetic correlations from GWA summary effect size statistics for each SNP, corrected for linkage disequilibrium between SNPs (Bulik-Sullivan et al. 2015). LDSC estimates of heritability and genetic correlations are similar to GCTA estimates, although GCTA estimates are generally more accurate (Evans et al. 2018; Ni et al. 2018). The advantage of LDSC is that it can be applied to published GWA summary statistics in contrast to GCTA which requires access to GWA data for individuals in the GWA study.

*Genomic Structural Equation Modeling (Genomic SEM).* In 2019, a third quantitative genomic analysis completed the arc from quantitative genetics to quantitative genomics by combining quantitative genetic structural equation model-fitting, routinely used in twin analyses, to LDSC heritabilities and genetic correlations (Grotzinger et al. 2019). Genomic SEM provides insights into the multivariate genetic architecture of cognitive traits (Grotzinger et al. 2019) and psychopathology (Grotzinger et al. 2022).

The second answer to the question about what to do with SNP associations that have such small effect sizes is the creation of polygenic scores.

### Polygenic Scores

A milestone that marks the spot where the DNA revolution began to transform the behavioral sciences is polygenic scores. Rather than using GWA genotypes to estimate SNP heritabilities and genetic correlations, polygenic scores use GWA genotypes to create a single score for each individual that aggregates, across all SNPs on a SNP chip, an individual’s genotype for each SNP (0, 1 or 2) weighted by the SNP’s effect size on the target trait as indicated by GWA summary statistics. In 2001, polygenic scores were introduced in plant and animal breeding (Meuwissen et al. 2001) and later in cognitive abilities (Harlaar et al. 2005) and psychopathology (Purcell et al. 2009). GWA summary statistics needed to create polygenic scores are now publicly available for more than 500 traits, including dozens for psychiatric disorders and other behavioral traits including cognitive traits (PGS Catalog 2022).

The most predictive polygenic scores in the behavioral sciences are for cognitive traits, especially educational attainment and intelligence. Early GWA studies of cognitive traits were underpowered to detect the small effects that we now know are responsible for heritability (Plomin and von Stumm 2018). In 2013, a landmark was a GWA study of educational attainment with a sample size exceeding 100,000 (Rietveld et al. 2013). A polygenic score derived from its GWA summary statistics predicted 2% of the variance of educational attainment in independent samples. The finding that the biggest effects accounted for only 0.0002 of the variance of educational attainment made it clear that much larger samples would be needed to scoop up more of the tiny effects responsible for the twin heritability estimate of about 40%. In the past decade, the predictive power of polygenic scores for educational attainment has increased with increasing sample sizes from 2% (Rietveld et al. 2013) to 5% (Okbay et al. 2016) to 10% (Lee et al. 2018) to 14% in a GWA study with a sample size of three million (Okbay et al. 2022). The current polygenic score for intelligence, derived from a GWA study with a sample of 280,000, predicted 4% of the variance (Savage et al. 2018), but, together, the polygenic scores for educational attainment and intelligence predicted 10% of the variance of intelligence test scores (Allegrini et al. 2019).

The next milestone will be to narrow the gap between heritability explained by polygenic scores and SNP heritability. A more daunting challenge will be to break through the ceiling of SNP heritability to reach the heritability estimated by twin studies. Reaching both of these destinations will be facilitated by even larger GWA studies and whole-genome sequencing (Wainschtein et al. 2022).

Polygenic scores are unique predictors because inherited DNA variations do not change systematically during life – there is no backward causation in the sense that nothing in the brain, behavior or environment changes inherited differences in DNA sequence. For this reason, polygenic scores can predict behavioral traits from early in life without knowing anything about the intervening pathways between genes, brain, and behavior.

Polygenic scores have brought behavioral genetics to the forefront of research in many areas of the life sciences because polygenic scores can be created in any sample of unrelated individuals for whom GWA genotype data are available. No special samples of twins or adoptees are needed, nor is it necessary to assess behavioral traits in order to use polygenic scores to predict them.

Although the implications and applications of polygenic scores derive from its power to predict behavioral traits without regard to explanation (Plomin and von Stumm 2022), another milestone on the road to behavioral genomics has been the leverage provided by GWA genotypes for causal modeling.

### Causal Modeling

A final milestone on the journey from behavioral genetics to behavioral genomics is a suite of new approaches that use GWA genotypes in causal models that attempt to dissect sources of genetic influence on behavioral traits (Pingault et al. 2018). Although traditional quantitative genetic models are causal models, GWA genotypes have enhanced causal modeling in research on assortative mating (Border et al. 2021; Yengo et al. 2018), population stratification (Abdellaoui et al. 2022; Lawson et al. 2020), and Mendelian randomization (Richmond and Davey Smith 2022).

An explosion of research on genotype-environment correlation was ignited by a 2018 paper in *Science* on the topic of the nature of nurture (Kong et al. 2018). The study included both parent and offspring GWA genotypes and showed that a polygenic score computed from non-transmitted alleles from parent to offspring influenced offspring educational attainment; these indirect effects were dubbed *genetic nurture*. GCTA has also been used to investigate genotype-environment correlation (Eilertsen et al. 2021). Although a great strength of behavioral genomics is its ability to investigate genetic influence in samples of unrelated individuals, combining GWA genotypes with traditional quantitative genetic designs has also enriched causal modeling (McAdams et al. 2022), for example, by comparing results within and between families (Brumpton et al. 2020; Howe et al. 2022).

## Conclusion

This whistle-stop tour has highlighted some of the milestones in a century of research in behavioral genetics. The progress is unmatched in the behavioral sciences and its discoveries have been transformative. The most exciting development is the synthesis of quantitative genetics and molecular genetics into behavioral genomics. The energy from this fusion will propel the field far into the future.
